# Early Prediction of Pembrolizumab-Induced Hypothyroidism Based on the Neutrophil-to-Lymphocyte Ratio

**DOI:** 10.7759/cureus.80049

**Published:** 2025-03-04

**Authors:** Yusuke Nakazawa, Ako Gannichida, Hirofumi Utsumi, Jun Araya, Takashi Kawakubo

**Affiliations:** 1 Department of Pharmacy, The Jikei University Hospital, Tokyo, JPN; 2 Department of Internal Medicine, The Jikei University School of Medicine, Tokyo, JPN

**Keywords:** early prediction, hypothyroidism, immune checkpoint inhibitor, immune-related adverse events, neutrophil-to-lymphocyte ratio, onset timing, pembrolizumab, predictive biomarker

## Abstract

Introduction

Pembrolizumab, an immune checkpoint inhibitor targeting programmed death-1 (PD-1)/PD-1 ligand (PD-L1), has demonstrated antitumor effects but can cause immune-related adverse events (irAEs) such as hypothyroidism. The neutrophil-to-lymphocyte ratio (NLR) may be linked to pembrolizumab efficacy and irAE risk. This study investigated the relationship between NLR trends and hypothyroidism onset, assessing its predictive potential.

Methods

This retrospective study analyzed 136 patients with advanced or recurrent cancer treated with pembrolizumab at The Jikei University Hospital, Tokyo, Japan, from February 2017 to September 2023. Patients were categorized based on hypothyroidism development, and their baseline NLR and time to treatment failure (TTF) were compared. NLR trends before hypothyroidism onset were also evaluated. Patients were further stratified based on onset timing (<90 days vs. ≥90 days), and NLR parameters prior to onset were also compared.

Results

Hypothyroidism occurred in 33 of 136 patients (24%). The hypothyroidism group had a significantly lower baseline NLR (P = 0.006) and longer TTF (P = 0.006). No significant NLR changes were observed before hypothyroidism onset (P = 0.626). However, the maximum NLR until onset was significantly lower in patients with early-onset hypothyroidism (<90 days) (P = 0.016). In contrast, no significant differences were observed in the mean and minimum NLR. A receiver-operating characteristic (ROC) analysis identified a maximum NLR cutoff of 4.3 for predicting early-onset hypothyroidism (P = 0.002, area under the curve (AUC) = 0.770).

Conclusions

A low baseline NLR was associated with hypothyroidism onset, and a persistently low NLR may contribute to early onset. These findings suggest that continuous NLR monitoring during pembrolizumab treatment may aid in predicting the risk of hypothyroidism and facilitate its appropriate management.

## Introduction

The immune checkpoint inhibitor pembrolizumab inhibits the binding of programmed death-1 (PD-1) and PD-1 ligand, thereby restoring and activating antigen-specific T cells against cancer cells to exert antitumor effects [1]. Pembrolizumab is used to treat a variety of cancers, including non-small cell lung cancer, renal cell carcinoma, malignant lymphoma, head and neck cancer, esophageal cancer, and malignant pleural mesothelioma. Although pembrolizumab has shown high therapeutic efficacy, it requires time for its effects to become apparent [2-4]. For patients who do not respond to treatment, alternative therapies must be considered, highlighting the need for the early identification of predictive markers of efficacy.

Pembrolizumab treatment may lead to immune-related adverse events (irAEs), including hypothyroidism [5]. The occurrence of irAEs may be linked to its treatment benefits [6-9]. Of these, hypothyroidism is a frequent irAE; however, the mechanism underlying its onset is unknown. Pembrolizumab activates immune cells to exert its antitumor effects; however, excessive activation may disrupt self-tolerance and contribute to the onset of hypothyroidism [10].

The neutrophil-to-lymphocyte ratio (NLR) has garnered attention in recent years as a potential predictor of pembrolizumab efficacy. A lower NLR at treatment initiation is associated with an improved treatment response [11-13]. This suggests that NLR may serve as a predictor not only of pembrolizumab efficacy but also for the risk of developing hypothyroidism. Our previous study on the PD-1 inhibitor nivolumab revealed that patients with lower NLR at treatment initiation tended to develop hypothyroidism earlier [14]. However, because the NLR fluctuates during treatment, relying solely on baseline NLR to predict the onset of hypothyroidism may be insufficient.

Currently, pembrolizumab is administered as monotherapy or in combination with other anticancer drugs. In this study, we continuously monitored NLR during pembrolizumab treatment to determine whether the timing of hypothyroidism onset can be predicted by evaluating the relationship between NLR fluctuations and the development of hypothyroidism.

## Materials and methods

Patient selection

This retrospective study included patients who received pembrolizumab treatment for advanced or recurrent cancer at The Jikei University Hospital, Tokyo, Japan, between February 2017 and September 2023. Patients were administered 200 mg pembrolizumab alone or in combination every three weeks for the treatment of unresectable advanced or recurrent non-small cell lung cancer, thymoma, urothelial carcinoma, renal cell carcinoma, head and neck cancer, esophageal cancer, endometrial cancer, ovarian cancer, or malignant lymphoma, for which pembrolizumab has been approved in Japan. The patients were identified through electronic medical records. Thyroid-stimulating hormone (TSH) and free thyroxine (FT4) levels were measured at the time of administration.

The exclusion criteria included patients with pre-existing hypothyroidism, a history of thyroid cancer, TSH or FT4 levels outside of the reference range at treatment initiation, and those who received combination therapy with lenvatinib, which is associated with a high incidence of hypothyroidism. In addition, patients who discontinued pembrolizumab after a single dose and those without regular laboratory measurements were excluded, as fluctuations in clinical values could not be analyzed. The reference values for TSH and FT4 levels were 0.34-4.04 µIU/mL and 0.88-1.67 ng/dL, respectively, based on the Japan Committee for Clinical Laboratory Standards. Hypothyroidism was defined as TSH levels exceeding the upper reference limit or FT4 falling below the lower reference limit on two consecutive occasions during pembrolizumab treatment. At the second measurement, patients with elevated TSH and low FT4 were classified as having overt hypothyroidism, whereas those with elevated TSH but normal FT4 levels were classified as having subclinical hypothyroidism.

NLR and hypothyroidism

The demographic characteristics, cancer type, treatment regimen, treatment duration, and laboratory data were extracted from electronic medical records. NLR was calculated by dividing the neutrophil count by the lymphocyte count from peripheral blood samples collected at each dose. Patients were classified into two groups based on the presence or absence of hypothyroidism and patient background (demographic data, NLR, etc.), treatment regimen, and time to treatment failure (TTF) at treatment initiation were then compared between the two groups. Based on previous studies, we adopted TTF in this study as a measure of treatment continuity [15,16]. TTF was defined as the time from the treatment initiation to the earliest event among death from any cause, disease progression, or treatment discontinuation (due to adverse effects or patient request). The decision to discontinue treatment was made by the attending physician based on disease progression or the occurrence of serious irAEs.

NLR fluctuation and onset time of hypothyroidism

To assess NLR fluctuations before the onset of hypothyroidism, NLR was analyzed at treatment initiation, at onset, and at three, six, and nine weeks before onset in patients who developed hypothyroidism. To determine the relationship between NLR and the onset time of hypothyroidism in more detail, patients were classified into two groups: those who developed hypothyroidism less than 90 days and those who developed it on day 90 or after. The onset of hypothyroidism was reported to be approximately three months [17-19]. Based on previous studies, the cutoff for the evaluation of the onset time in this study was set at 90 days. The mean, minimum, and maximum NLR until onset were compared between the two groups, and the cutoff value of NLR to predict the onset of hypothyroidism was calculated using receiver-operating characteristic (ROC) analysis.

Statistics

The distribution of continuous variables was evaluated by the Shapiro-Wilk test. For comparisons of unpaired continuous variables, the t-test was used for normally distributed data, while the Mann-Whitney U test was used for non-normally distributed data. Categorical variables were statistically analyzed using Fisher’s exact test. TTF was evaluated by the Kaplan-Meier method and compared using the log-rank test. To compare changes in NLR over time, we used the Kruskal-Wallis test for non-normally distributed data. ROC analysis was used to determine the optimal NLR cutoff value that predicted hypothyroidism. All statistical analyses were performed using BellCurve for Excel (Social Survey Research Information Co. Ltd., Tokyo, Japan) with a P value of < 0.05 indicating statistical significance.

Ethics approval

The study procedures were conducted in accordance with the ethical standards of the institutional and/or national research committee, as well as the 1964 Declaration of Helsinki and its later amendments or comparable ethical standards. This study was approved by the Ethics Committee of The Jikei University School of Medicine (Approval No. 34-449(11606)). Informed consent for study participation and data usage was obtained from all patients using an opt-out approach.

## Results

Comparison of the patient background data

A total of 136 patients were analyzed. Pembrolizumab was primarily administered every three weeks, although in some cases, it was given temporarily every four weeks because of hospital closures or patient requests. The median (range) follow-up period of the patients was 199 (21-1583) days. During the observation period, 33 patients (24%) developed hypothyroidism. At the onset of hypothyroidism, the mean TSH level was 16.41 ± 25.20 µIU/mL, and the mean FT4 level was 0.90 ± 0.36 ng/dL. Among these patients, 12 had overt hypothyroidism, while 21 had subclinical hypothyroidism (Table 1). The cancer types and treatment regimens for the patients treated with pembrolizumab are listed in Table 2. No significant differences were observed in cancer type or treatment regimen between patients who developed hypothyroidism (n = 33) and those who did not (n = 103). Table 3 presents the patient characteristics and laboratory values at treatment initiation. The mean NLR was 3.57 ± 1.83 in patients who developed hypothyroidism and 5.25 ± 5.03 in those who did not, with a significantly lower NLR in the hypothyroidism group (P = 0.006). The mean neutrophil counts were also significantly lower in the hypothyroidism group (4443 ± 1559 vs. 5272 ± 2433/µL, P = 0.026).

**Table 1:** Laboratory data at the second measurement (hypothyroidism onset) TSH: thyroid-stimulating hormone; FT4: free thyroxine Data are expressed as mean ± SD.

TSH (µIU/mL)	FT4 (ng/dL)	Hypothyroidism (overt/subclinical)
16.41 ± 25.20	0.90 ± 0.36	12/21

**Table 2:** Characteristics of the treatment regimens a) χ² test; b) Fisher’s exact test

Treatment regimen	Hypothyroidism (n = 33)	Non-hypothyroidism (n = 103)	χ² value	P value	
Non-small cell lung cancer					
Monotherapy	5	17	0.00	1.000	^a)^
+ cisplatin + pemetrexed	2	8	N/A	1.000	^b)^
+ carboplatin + pemetrexed	0	8	N/A	0.198	^b)^
+ carboplatin + nab-paclitaxel	2	8	N/A	1.000	^b)^
+ carboplatin + paclitaxel	1	2	N/A	0.569	^b)^
+ necitumumab	1	2	N/A	0.569	^b)^
Thymoma					
Monotherapy	0	1	N/A	1.000	^b)^
Urothelial cancer					
Monotherapy	9	31	0.01	0.928	^a)^
Renal cell cancer					
+ axitinib	6	8	1.92	0.087	^a)^
Head and neck cancer					
Monotherapy	5	6	1.80	0.179	^a)^
+ cisplatin + fluorouracil	1	6	N/A	1.000	^b)^
Esophageal cancer					
+ cisplatin + fluorouracil	1	2	N/A	0.569	^b)^
Endometrial cancer					
Monotherapy	0	2	N/A	1.000	^b)^
Ovarian cancer					
Monotherapy	0	1	N/A	1.000	^b)^
Malignant lymphoma					
Monotherapy	0	1	N/A	1.000	^b)^

**Table 3:** Characteristics of the patients AST: aspartate aminotransferase; ALT: alanine aminotransferase; TSH: thyroid-stimulating hormone; FT4: free thyroxine; FT3: free triiodothyronine; df: degrees of freedom a) Welch's t-test; b) χ² test; data are expressed as mean ± SD.

Variables	Hypothyroidism (n = 33)	Non-hypothyroidism (n = 103)	t value	df	χ² value	P value	
Age	69.0 ± 13.3	69.1 ± 11.5	0.05	48.17	N/A	0.962	^a)^
Gender (male/female)	27/6	83/20	N/A	N/A	0.02	0.875	^b)^
Body weight (kg)	58.4 ± 14.1	54.5 ± 9.7	1.47	41.88	N/A	0.149	^a)^
Body surface area (/m^2^)	1.64 ± 0.21	1.59 ± 0.15	1.41	43.04	N/A	0.167	^a)^
Laboratory data							
AST	19.8 ± 7.8	22.4 ± 13.9	1.33	97.87	N/A	0.185	^a)^
ALT	17.0 ± 13.0	18.4 ± 16.2	0.51	65.64	N/A	0.615	^a)^
Blood urea nitrogen	16.3 ± 10.3	18.1 ± 6.8	0.92	41.16	N/A	0.361	^a)^
Serum creatinine	1.26 ± 1.90	1.06 ± 0.65	0.59	34.42	N/A	0.561	^a)^
Serum albumin (g/dL)	3.89 ± 0.51	3.70 ± 0.47	1.85	50.94	N/A	0.070	^a)^
C-reactive protein (mg/dL)	1.59 ± 3.29	3.05 ± 4.48	1.96	72.47	N/A	0.054	^a)^
TSH (µIU/mL)	2.40 ± 0.90	2.07 ± 0.93	1.80	54.89	N/A	0.078	^a)^
FT4 (ng/dL)	2.36 ± 0.32	2.25 ± 0.37	1.32	50.64	N/A	0.193	^a)^
FT3 (pg/mL)	1.22 ± 0.22	1.28 ± 0.20	1.57	61.10	N/A	0.122	^a)^
Neutrophil count (/µL)	4443 ± 1559	5272 ± 2433	2.26	84.17	N/A	0.026	^a)^

Hypothyroidism and treatment duration

**Figure 1:**
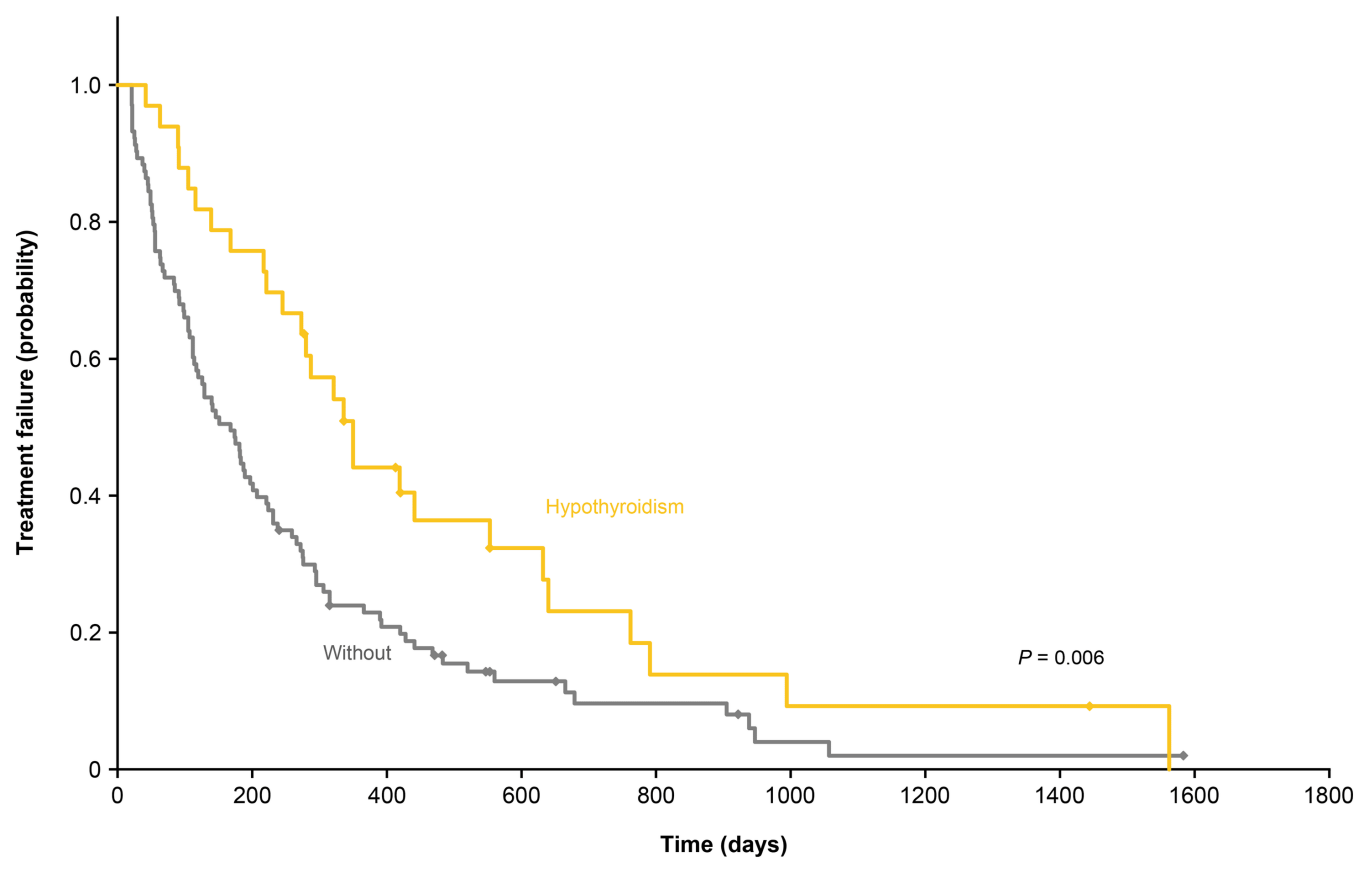
Time to treatment failure of patients with or without hypothyroidism

The TTF in patients with and without hypothyroidism is shown in Figure 1. The median (range) TTF was 336 (42-1562) days in patients with hypothyroidism, which was significantly longer compared with 138 (21-1583) days in those without hypothyroidism (P = 0.006).

NLR fluctuation before the onset of hypothyroidism

**Figure 2:**
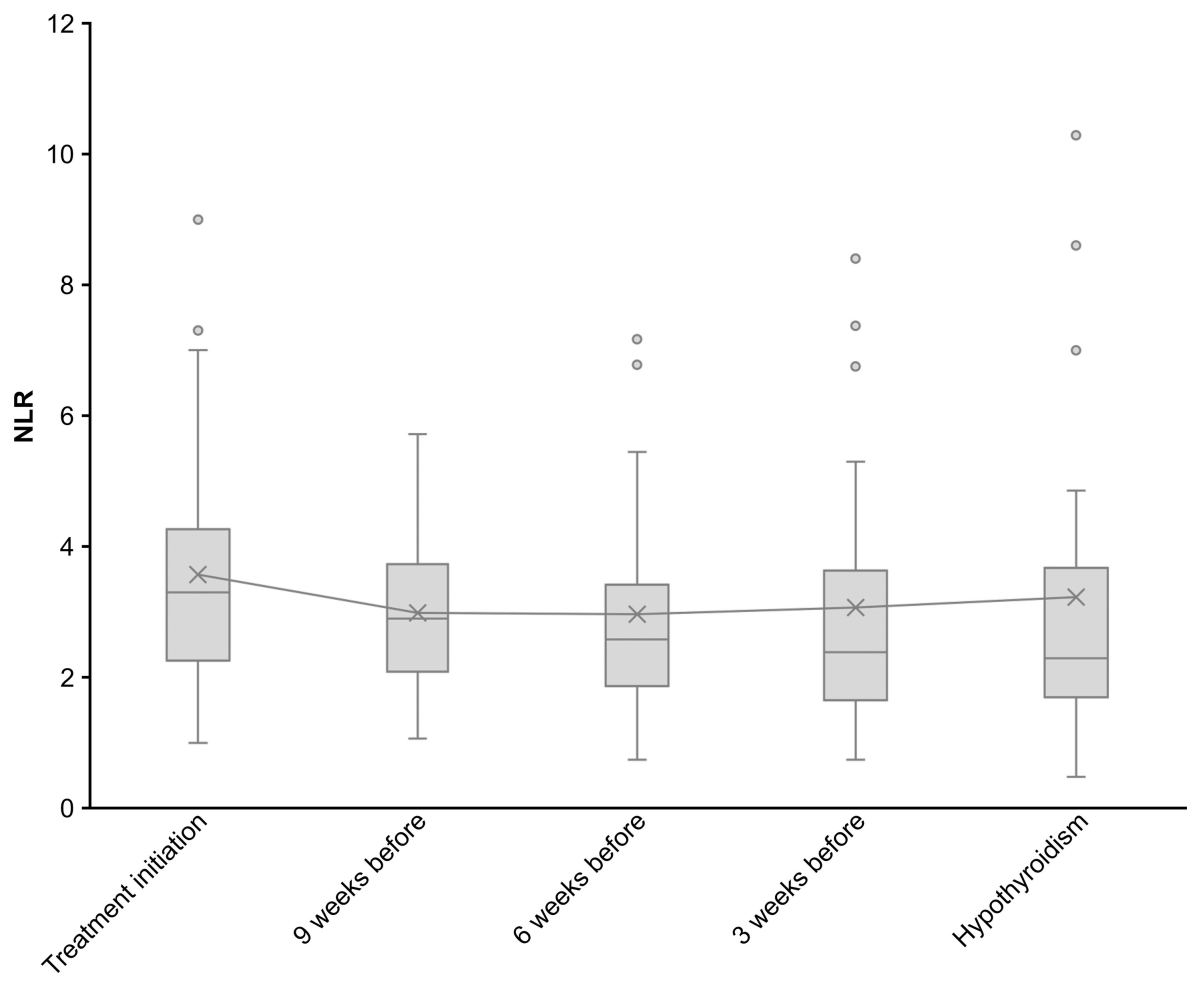
Changes in NLR from treatment initiation to hypothyroidism onset NLR: neutrophil-to-lymphocyte ratio

The fluctuation of NLR over time in patients who developed hypothyroidism at treatment initiation, onset, and at three, six, and nine weeks before onset is shown in Figure 2. In the hypothyroidism group, the mean NLR before treatment initiation was 3.57 ± 1.83. At onset, it was 3.23 ± 2.43. At three, six, and nine weeks before onset, it was 3.07 ± 1.93, 2.97 ± 1.56, and 2.99 ± 1.22, respectively. The Kruskal-Wallis test was performed to compare NLR values at different time points, and the analysis showed no significant differences (χ²(4) = 4.07, P = 0.397).

NLR in patients who developed hypothyroidism before and after day 90

**Figure 3:**
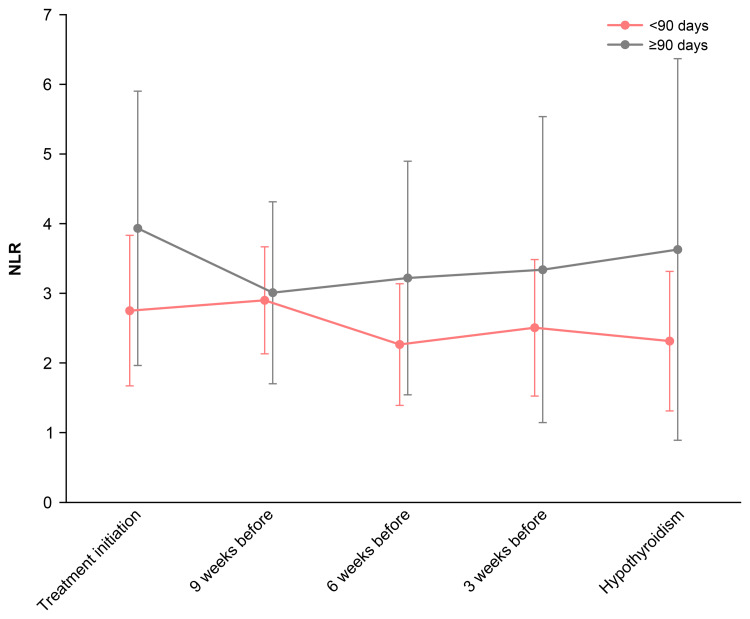
Changes in NLR by hypothyroidism onset timing (<90 days vs. ≥90 days) NLR: neutrophil-to-lymphocyte ratio

**Figure 4:**
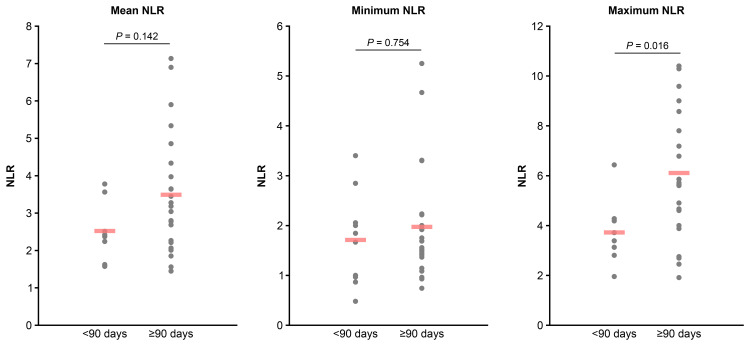
Comparison of NLR between hypothyroidism onset before and after day 90 (<90 days vs. ≥90 days) NLR: neutrophil-to-lymphocyte ratio

**Figure 5:**
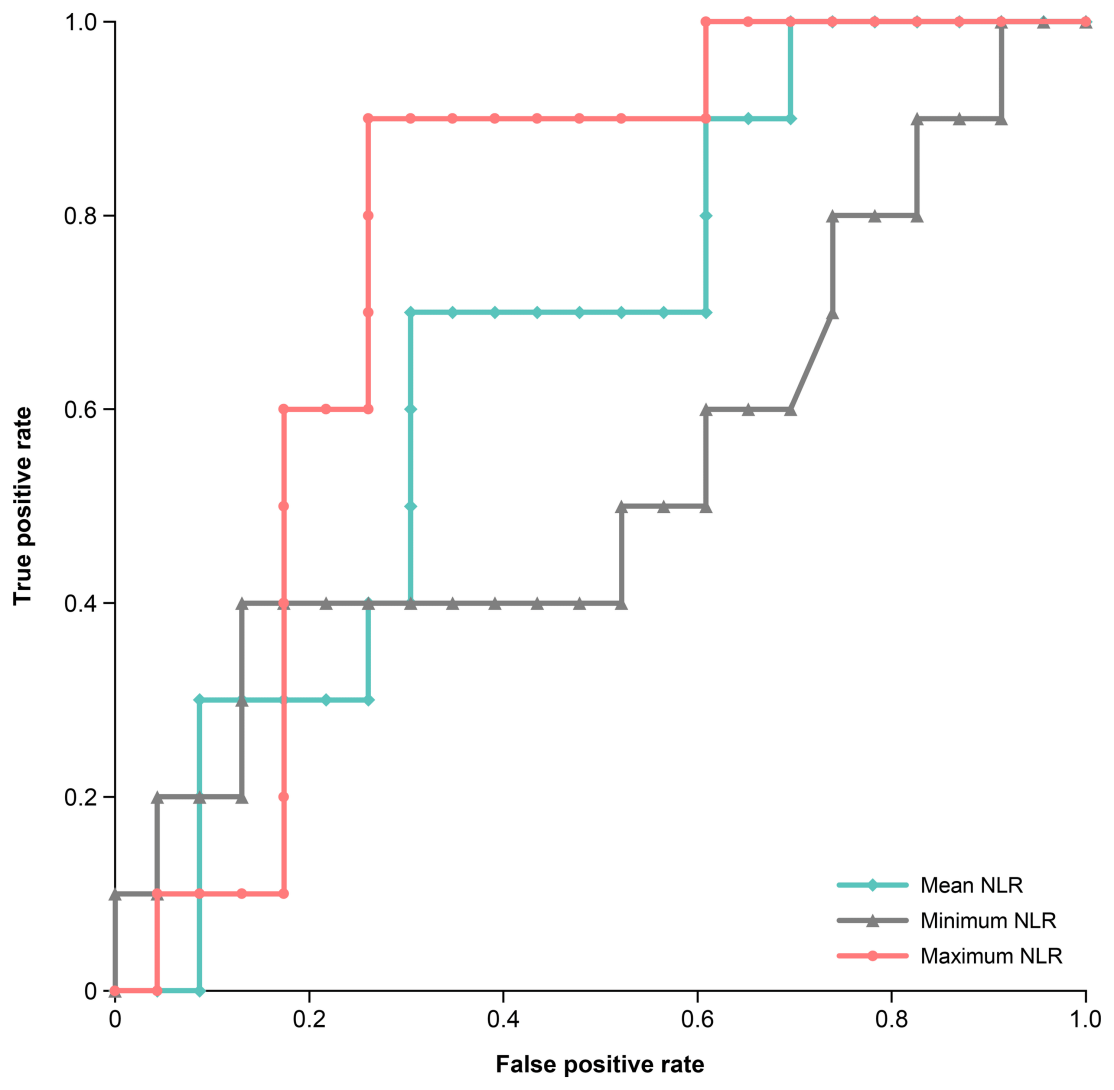
Optimal NLR cut-off value for predicting hypothyroidism: ROC analysis The curve illustrates the tradeoff between the model‘s sensitivity and specificity. The areas under the ROC for each NLR parameter were plotted. The AUC for mean NLR was 0.665 (P = 0.098), for minimum NLR was 0.537 (P = 0.764), and for maximum NLR was 0.770 (P = 0.002). The cutoff value in the ROC curve for maximum NLR was 4.3, with a sensitivity and specificity of 90% and 74%. NLR: neutrophil-to-lymphocyte ratio; ROC: receiver operating characteristics curves; AUC: area under the curve

Of the 33 patients who developed hypothyroidism, 10 (30%) developed it in less than 90 days, whereas 23 (70%) developed it on day 90 or after. Figure 3 shows the NLR trends in both groups, indicating consistently lower NLR values were observed in those who developed hypothyroidism in less than 90 days. Figure 4 presents a comparison of the NLR values until the onset of hypothyroidism. The mean NLR was 2.52 ± 0.80 for patients who developed hypothyroidism in less than 90 days and 3.49 ± 1.57 in those who developed it on day 90 or after (P = 0.142). The minimum NLR values were 1.71 ± 0.88 and 1.97 ± 1.12, respectively (P = 0.754), whereas the maximum NLR values were 3.72 ± 1.14 and 6.11 ± 2.60, respectively. The maximum NLR values were significantly lower in patients who developed hypothyroidism in less than 90 days (P = 0.016). ROC analysis identified a maximum NLR cutoff value of 4.3 for predicting early-onset hypothyroidism (less than 90 days) (P = 0.002, area under the curve (AUC) = 0.770), with a sensitivity of 90% and a specificity of 74% (Figure 5); however, no statistically significant cutoff values were observed for the mean and minimum NLR (P = 0.098, AUC = 0.665 and P = 0.764, AUC = 0.537, respectively). Fisher’s exact test demonstrated that having a maximum NLR below 4.3 before the onset of hypothyroidism was a significant risk factor for developing hypothyroidism in less than 90 days (odds ratio: 25.5 (95% CI, 2.65-245.84), P < 0.001).

## Discussion

In this study, we examined the relationship between NLR and the onset of hypothyroidism during pembrolizumab treatment. Patients who developed hypothyroidism had a significantly longer TTF than those who did not. This is consistent with previous studies showing that patients who develop irAEs have prolonged TTF [15,16]. The patients in this study exhibited similar characteristics to those described in previous reports, suggesting that the results are generalizable. In addition, we compared the NLR at treatment initiation in patients who developed hypothyroidism compared with those who did not. The hypothyroidism group had a significantly lower NLR. The relationship between NLR and treatment efficacy has been reported previously, with lower NLR being linked to better treatment outcomes [20-22]. Furthermore, since the development of irAEs is closely related to immune activation, NLR evaluation at treatment initiation may serve not only as a predictor of treatment response but also as a useful tool for assessing the risk of hypothyroidism. There was no significant difference in lymphocyte counts at treatment initiation between the groups that developed and did not develop hypothyroidism; however, the neutrophil count was significantly lower in the hypothyroidism group. This suggests that although the absolute lymphocyte count did not change in the hypothyroidism-developed group, the relative proportion of lymphocytes was increased because of the lower neutrophil count. This immune environment may promote T-cell activation by PD-1 inhibitors, which may contribute to the development of irAEs, including hypothyroidism.

In this study, hypothyroidism was more frequently subclinical rather than overt at the time of onset. Since mild hypothyroidism often presents as subclinical, with only elevated TSH and normal FT4 levels, our findings suggest that NLR monitoring may help predict the early stages of pembrolizumab-induced hypothyroidism.

To evaluate the association between NLR fluctuations and the development of hypothyroidism, we assessed NLR fluctuations at treatment initiation, at the onset of hypothyroidism, and at three, six, and nine weeks before the onset of hypothyroidism in patients who developed hypothyroidism. No significant differences were found in these fluctuations. A previous study on irAEs and NLR showed that while NLR increases until the onset of interstitial pneumonia, no significant change occurs until the onset of endocrine irAEs, such as thyroiditis and hypophysitis [23]. Similarly, our study found no significant NLR fluctuations before hypothyroidism onset, suggesting that hypothyroidism shares characteristics with endocrine irAEs. These findings highlight the need for symptom-specific evaluation when predicting irAE onset based on NLR. Our results indicate that patients who developed hypothyroidism already had a low NLR at treatment initiation, which remained stable until onset. Thus, predicting hypothyroidism onset solely from pre-onset NLR changes may be challenging.

Furthermore, when evaluating the relationship between hypothyroidism onset timing and NLR, we found that the maximum NLR until onset was significantly lower in patients who developed hypothyroidism in less than 90 days; however, no significant differences were observed in the mean or minimum NLR values. In addition, ROC analysis identified a maximum NLR of 4.3 as an important threshold for predicting early-onset hypothyroidism (in less than 90 days), but no statistically significant cutoff values were obtained for the mean or minimum NLR. The continued maintenance of low NLR values is associated with favorable treatment outcomes [21,22]. These results suggest that an NLR that remains persistently low, as opposed to one that is temporarily low, may be a factor in the early development of hypothyroidism. Therefore, continuous monitoring of the NLR is important for assessing the risk of developing hypothyroidism during pembrolizumab treatment.

This study had several limitations. First, it was a single-center retrospective study, and a detailed analysis by cancer type was not conducted. Second, due to the limited sample size, validation in a larger population is necessary. Third, a detailed analysis by cancer type is necessary because TTF may vary depending on the type and stage of cancer. Fourth, before the onset of hypothyroidism, some patients may first experience destructive thyroiditis or transient hyperthyroidism. However, these conditions were not evaluated in this study. To accurately assess the onset of hypothyroidism, the potential effects of destructive thyroiditis and transient hyperthyroidism should be considered in future studies. Further clinical prospective trials and multicenter collaborative studies will be needed to overcome these challenges.

The presence of anti-thyroid peroxidase antibodies or anti-thyroglobulin antibodies is associated with the development of hypothyroidism [19,24]; however, these test data are not routinely measured in clinical practice. On the other hand, because neutrophil and lymphocyte counts are routinely measured, NLR may serve as a convenient and practical predictive marker for evaluating hypothyroidism associated with pembrolizumab treatment.

## Conclusions

This study revealed that patients with low NLR at treatment initiation and during treatment are more likely to develop hypothyroidism. In particular, the maximum NLR until the onset was significantly lower in patients who developed hypothyroidism in less than 90 days. Moreover, a maximum NLR until the onset of less than 4.3 may serve as a threshold for predicting the early onset of hypothyroidism. These findings indicate that regular monitoring of NLR during pembrolizumab treatment may help predict the risk of hypothyroidism. Continuous thyroid function monitoring is particularly important in patients with persistently low NLR, as they may be at increased risk of early-onset hypothyroidism.
